# Bimekizumab for psoriasis treatment: A Canadian real-world multicenter study

**DOI:** 10.1016/j.jdin.2025.04.011

**Published:** 2025-06-06

**Authors:** Fiona Lovegrove, Rachel Asiniwasis, Natalie Cunningham, Jennifer Lipson, Ashley O’Toole, Kerri Purdy, Ashley Sutherland, Kirsten Walker, Melinda Gooderham

**Affiliations:** a Lovegrove Dermatology, London, Ontario, Canada; b Department of Medicine, Schulich School of Medicine and Dentistry, London, Ontario, Canada; c Origins Dermatology Centre, Regina, Saskatchewan, Canada; d Maritime Dermatology, Halifax, Nova Scotia, Canada; e Probity Medical Research, Waterloo, Ontario, Canada; f Division of Dermatology, The Ottawa Hospital, Ottawa, Ontario, Canada; g Department of Medicine, University of Ottawa, Ottawa, Ontario, Canada; h West Ottawa Specialty Care, Ottawa, Ontario, Canada; i Division of Dermatology and Rheumatology, Children’s Hospital of Eastern Ontario, Ottawa, Ontario, Canada; j SKiN Health, Peterborough, Ontario, Canada; k Department of Medicine, Dalhousie University, Halifax, Nova Scotia, Canada; l Division of Dermatology, Department of Medicine, University of Saskatchewan, Saskatoon, Saskatchewan, Canada; m Walker Dermatology, Saskatoon, Saskatchewan, Canada; n Queen’s University, Kingston, Ontario, Canada

**Keywords:** bimekizumab, biologics, clinical practice, IL-17 inhibitor, psoriasis, real-world data

*To the Editor:* Biologic therapies are pivotal in the management of moderate-to-severe psoriasis and psoriatic arthritis. Bimekizumab (Bimzelx, UCB), a monoclonal antibody targeting interleukin-17A and interleukin-17F, is relatively new to the Canadian biologic market and published real-world data remains limited.

We conducted a retrospective chart review of 154 patients with moderate-to-severe plaque psoriasis treated with bimekizumab at seven outpatient dermatology clinics across Canada (data cut-off: January 10, 2024). The mean patient age was 53.9 years, and 75 patients (49%) were female. The most common comorbidity was psoriatic arthritis (62 patients; 40%), and 113 patients (73%) had psoriasis at ≥1 high impact site. Most patients (84%) were on a Q8W maintenance schedule. The median treatment duration was 8.5 (range: 0.4-68.3) months. At the data cut-off, 130 patients (84%) remained on bimekizumab.

Psoriasis improved with bimekizumab in almost all patients from a baseline mean (SD) Psoriasis Area and Severity Index (PASI) score of 10.8 (8.0). PASI75, PASI90, and PASI100 rates were 72%, 60% and 47%, respectively at the latest assessment (Fig 1). The proportion of patients with PASI scores of ≤2 increased from 7% at baseline to 76% at latest assessment (Fig 1). Bionaïve patients showed a trend toward higher PASI responses than bioexperienced patients, but differences were not statistically significant. The difference in PASI scores between randomized clinical trials and our analysis may be due to the higher proportion of bioexperienced patients in our sample (31% to 44% vs 71%), indicating a more difficult-to-treat population.^1^, ^2^, ^3^, ^4^

**Fig 1:**
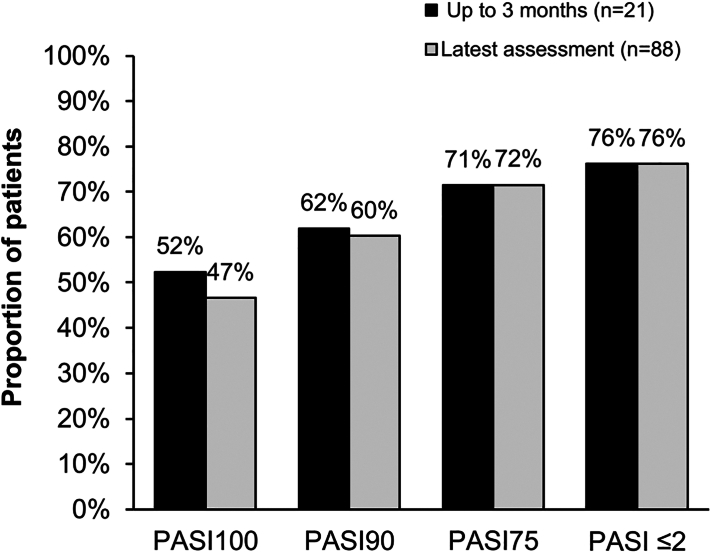
PASI scores at up to 3 months of treatment and at the latest assessment analysis of PASI scores in the cohort. Data are means. Proportion of patients who achieved PASI100, PASI 90, PASI75, and PASI ≤2 with available PASI assessments at up to 3 month time point and at latest assessment out of all patients with available data at that time point. *PASI*, Psoriasis Area and Severity Index.

The mean Dermatology Life Quality Index (DLQI) score at baseline was 19.4, indicating severe quality of life (QOL) impairment and improved significantly with treatment (mean latest DLQI 10). Baseline DLQI scores in our study were higher than those reported in the randomized clinical trials, representing a potential bias to QOL data from more severely impacted patients. There was a strong correlation between PASI and DLQI improvement (0.716, *P* < .001; *n* = 18).

Bimekizumab was well-tolerated, with no new safety signals over 132 patient-years. Thirty-six patients (23%) had oral candidiasis and 8 patients experienced recurrent/ongoing oral candidiasis.

Twenty-four patients (16%) discontinued treatment. The difference in the rate of bimekizumab discontinuation in our analysis and clinical studies (∼6%) likely reflects the availability of other targeted treatments, allowing patients to switch if outcome targets are not timely met. There was a strong correlation between a high DLQI at latest assessment and treatment discontinuation (r = 0.7813, *P* < .0001; *n* = 22; Fig 2). Patients who discontinued tended to have more severe disease at final assessment than those still on treatment (mean latest PASI score 9.5 vs 1.1; r = 0.5244, *P* < .0001; *n* = 88). Our analysis suggests that improvement in QOL, and to a lesser extent in clinical signs of psoriasis, played a significant role in treatment continuation. This reinforces that both patient- and physician-reported outcomes should influence the optimal treatment regimen.

**Fig 2:**
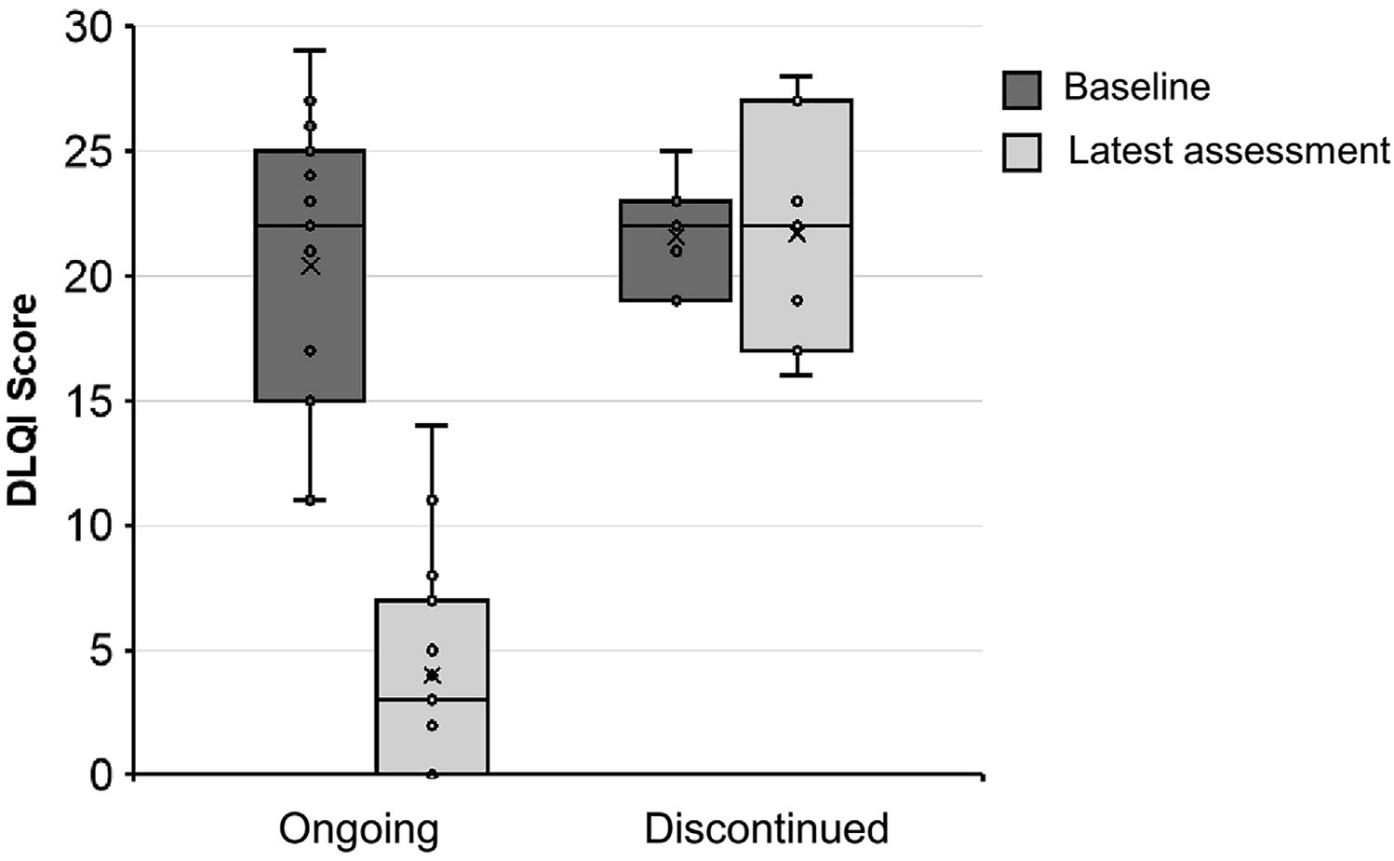
DLQI scores in ongoing and discontinued patients Box plot of DLQI scores at baseline and latest assessment for patients who remain on bimekizumab treatment and those who discontinued. The *centre line* denotes the median value (50th percentile), the x denotes the mean value, and the *circles* denote each individual data point. The *boxes* contain the 25th to 75th percentiles of dataset. The whiskers mark the minimum and maximum values. *DLQI*, Dermatology Life Quality Index.

Limitations of this study include its retrospective nature and the variability of clinical practice and assessments.

We showed that the results from randomized controlled clinical trials translate well into the real-world clinical practice, as represented by a large cohort of Canadian patients with psoriasis, treated with bimekizumab for up to 5 years.

## Conflicts of interest

Dr Lovegrove has been an investigator for/conducted clinical research sponsored by Abbvie, Bausch Health, COREvitas, GSK, Incyte, Janssen, LEO Pharma, UCB; and a speaker or consultant for: Abbvie, Amgen, Bausch Health, Celgene, Cipher, Galderma, Janssen, LEO Pharma, Eli Lilly, Novartis, Pfizer, Sanofi-Genzyme, and Valeant. Dr Gooderham has been an investigator, speaker and/or advisor for: AbbVie, Acelyrin, Amgen, Akros, Arcutis, Aristea, AnaptysBio, Apogee, Bausch Health, BMS, Boehringer Ingelheim, Cara, Dermira, Dermavant, Eli Lilly, Galderma, GSK, Incyte, InMagene, JAMP Pharma, Janssen, Kyowa Kirin, LEO Pharma, MedImmune, Meiji, Merck, Moonlake, Nimbus, Novartis, Pfizer, Regeneron, Roche, Sanofi Genzyme, Sun Pharma, Tarsus, Takeda, UCB, Union, and Ventyx. Dr Asiniwasis has been an investigator, speaker, and/or advisor for: AbbVie, Arcutis, Apogee, Bausch Health, BMS, Boehringer Ingelheim, Eli Lilly, Galderma, Incyte, Janssen, LEO Pharma, L’Oreal, Novartis, Pfizer, Sanofi-Regeneron, Sun Pharma, UCB, and Valeant. Dr Sutherland has been an advisor/speaker for AbbVie, Arcutis, Bausch Health, BMS, Boehringer Ingelheim, Eli Lilly, Galderma, Incyte, Janssen, LEO Pharma, L’Oreal, Novartis, Pfizer, Sanofi, and Sun Pharma. Dr Cunningham has been a consultant and/or speaker for AbbVie, Bausch Health, Eli Lilly, Novartis, Pfizer, Sun Pharma, UCB, Bristol-Myers Squibb, LEO Pharma, Janssen, Arcutis, Boehringer Ingelheim, and Incyte. Dr O’Toole has been a consultant and/or speaker for Janssen, Arcutis, Sun Pharma, AbbVie, Galderma, Eli Lilly, LEO Pharma, Boehringer Ingelheim, Bristol-Myers Squibb, and Miravo. Dr Purdy has been a consultant and/or speaker for AbbVie, Arcutis, Boehringer Ingelheim, Bausch Health, Bristol-Myers Squibb, Eli Lilly, Galderma, Incyte, LEO Pharma, Mallinckroft, Novartis, Pfizer, Sanofi, Sun Pharma, UCB, Janssen, and is on the Board of Directors of and is a committee member of the Canadian Dermatology Association. Dr Walker has no conflicts of interest to declare. Dr Lipson has been a consultant and/or speaker for AbbVie, Bausch Health, Beiersdorf, Galderma, JAMP, Janssen, LEO Pharma, Eli Lilly, Novartis, Pfizer, Sanofi, Sun Pharma, and UCB; has received support for attending meetings from JAMP, Janssen, Sun Pharma, and UCB; has served on advisory boards for AbbVie, Bausch Health, Beiersdorf, Galderma, Janssen, LEO Pharma, Eli Lilly, Novartis, Pfizer, Sanofi, Sun Pharma, and UCB; and is on the Medical Advisory Committee for Acne and Rosacea Society of Canada.
